# Thoracoscopic right S1 + S3 segmentectomy for lung cancer with a displaced B2 bronchus: a case report

**DOI:** 10.1186/s44215-026-00261-6

**Published:** 2026-04-23

**Authors:** Yuki Shindo, Shinjiro Nagai, Katsushi Toyohara, Kaoru Ochi, Kiyoshi Sato, Nobuharu Hanaoka, Takahiro Katsumata

**Affiliations:** 1 Department of Thoracic Surgery, Takatsuki Red Cross Hospital, 1-1-1 Abuno, Takatsuki, Osaka, 569-1096 Japan; 2 Department of Thoracic and Cardiovascular Surgery, Osaka Medical and Pharmaceutical University, Osaka, Japan; 3 Department of Thoracic Surgery, Hirakata City Hospital, Osaka, Japan; 4 Department of Thoracic Surgery, Hokusetsu General Hospital, Osaka, Japan

**Keywords:** Lung cancer, Bronchial anatomy, Segmentectomy, Displaced right upper bronchus, Video-assisted thoracoscopic surgery (VATS), Three-dimensional computed tomography bronchography and angiography (3D-CTBA), Case report

## Abstract

**Background:**

Tracheobronchial branching anomalies are uncommon, but increase the risk of intraoperative vascular or bronchial injury if not recognized preoperatively. An independent origin of the right B2 bronchus from the bronchus intermedius is particularly rare and may be accompanied by complex pulmonary venous and arterial variations. Careful preoperative anatomical assessment is crucial when planning anatomical segmentectomy in such cases.

**Case presentation:**

A 73-year-old woman was referred to our hospital after a 12-mm partially solid nodule with a cavity at the border between segments S1 and S3 in the right upper lobe was incidentally detected on chest computed tomography (CT). Transbronchial biopsy confirmed squamous cell carcinoma. Bronchoscopy and coronal chest CT showed a displaced right B2 bronchus arising from the bronchus intermedius, with a common B1 + 3 bronchus arising from the right main bronchus. Contrast-enhanced three-dimensional CT bronchography and angiography revealed a complex venous pattern, with V1b draining into the superior pulmonary vein and two additional veins from segment S1 draining into the central vein, as well as a so-called top pulmonary vein formed by a portion of V2 draining into V6. The A3a branch arose from the interlobar pulmonary artery, and no recurrent A2 branch was present. The patient underwent three-port video-assisted thoracoscopic surgery of right S1 + S3 segmentectomy with hilar lymph node dissection. Because the lesion was small and clinically early stage, anatomical segmentectomy was considered feasible provided that an adequate surgical margin could be secured. Despite incomplete fissures between the upper and middle and between the upper and lower lobes, the operation was completed safely by following the preoperative three-dimensional vascular and bronchial roadmap, with careful hilar dissection and indocyanine green-guided identification of the intersegmental plane. The postoperative course was uneventful. The final pathological diagnosis was pT1bN0M0 stage IA2 squamous cell carcinoma, with a pathological surgical margin of 2.7 cm.

**Conclusions:**

This case shows that detailed preoperative three-dimensional assessment of the bronchovascular anatomy, including displaced bronchi and atypical pulmonary veins, can facilitate safe anatomical segmentectomy in the presence of rare tracheobronchial anomalies. Such planning is important to avoid vascular injury and to secure adequate surgical margins in segmentectomy for early-stage lung cancer.

**Supplementary Information:**

The online version contains supplementary material available at 10.1186/s44215-026-00261-6.

## Background

Tracheobronchial branching anomalies are relatively rare, with a reported incidence of 0.64–0.76% [1, 2]. Among these variants, an independent origin of the right B2 bronchus from the bronchus intermedius is extremely rare, having been reported in only 0.1% of cases [2]. Because such anatomical variants increase the risk of intraoperative injury, meticulous preoperative planning using bronchoscopy and contrast-enhanced three-dimensional computed tomography (CT) bronchography and angiography (3D-CTBA) is crucial. Here, we report a case of video-assisted thoracoscopic surgery (VATS) right S1 + S3 segmentectomy for lung cancer in a patient with a displaced bronchus and complex vascular anomalies, in whom meticulous preoperative assessment enabled safe resection while preserving oncological adequacy. This case report was prepared in accordance with the SCARE 2025 guideline [3].

## Case presentation

**Fig. 1:**
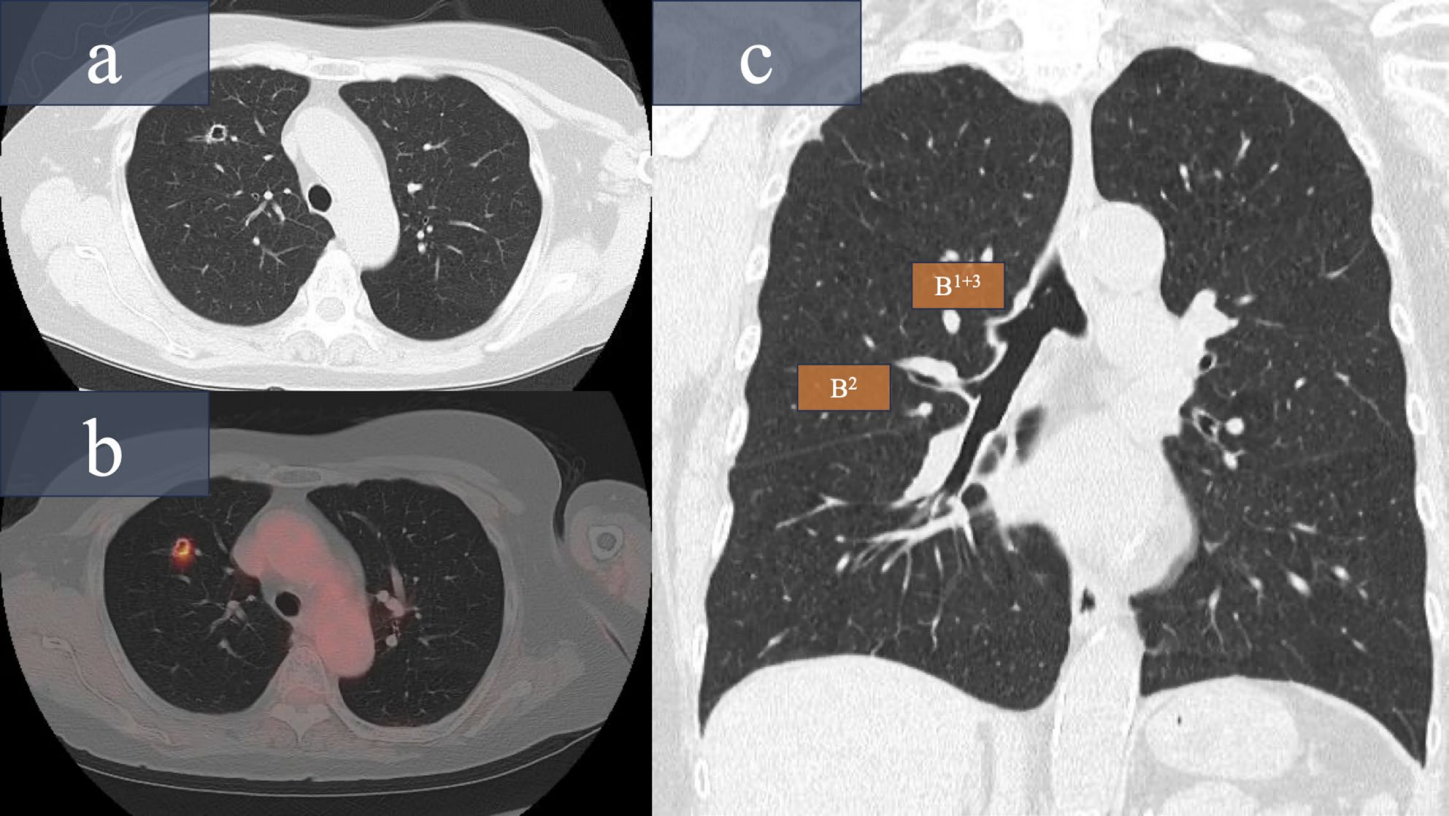
Preoperative imaging findings. **a** Computed tomography (CT) showing a 12-mm partially solid nodule with a cavity in the right upper lobe at the border between segments S1 and S3. **b** ^18^F-fluorodeoxyglucose positron emission tomography/CT (¹⁸F-FDG PET/CT) showing FDG uptake in the nodule, with SUV_max_ of 7.4. **c** Coronal chest CT showing the displaced B2 bronchus arising from the bronchus intermedius, whereas the B1 + 3 bronchus (right upper bronchus) originated from the right main bronchus

**Fig. 2:**
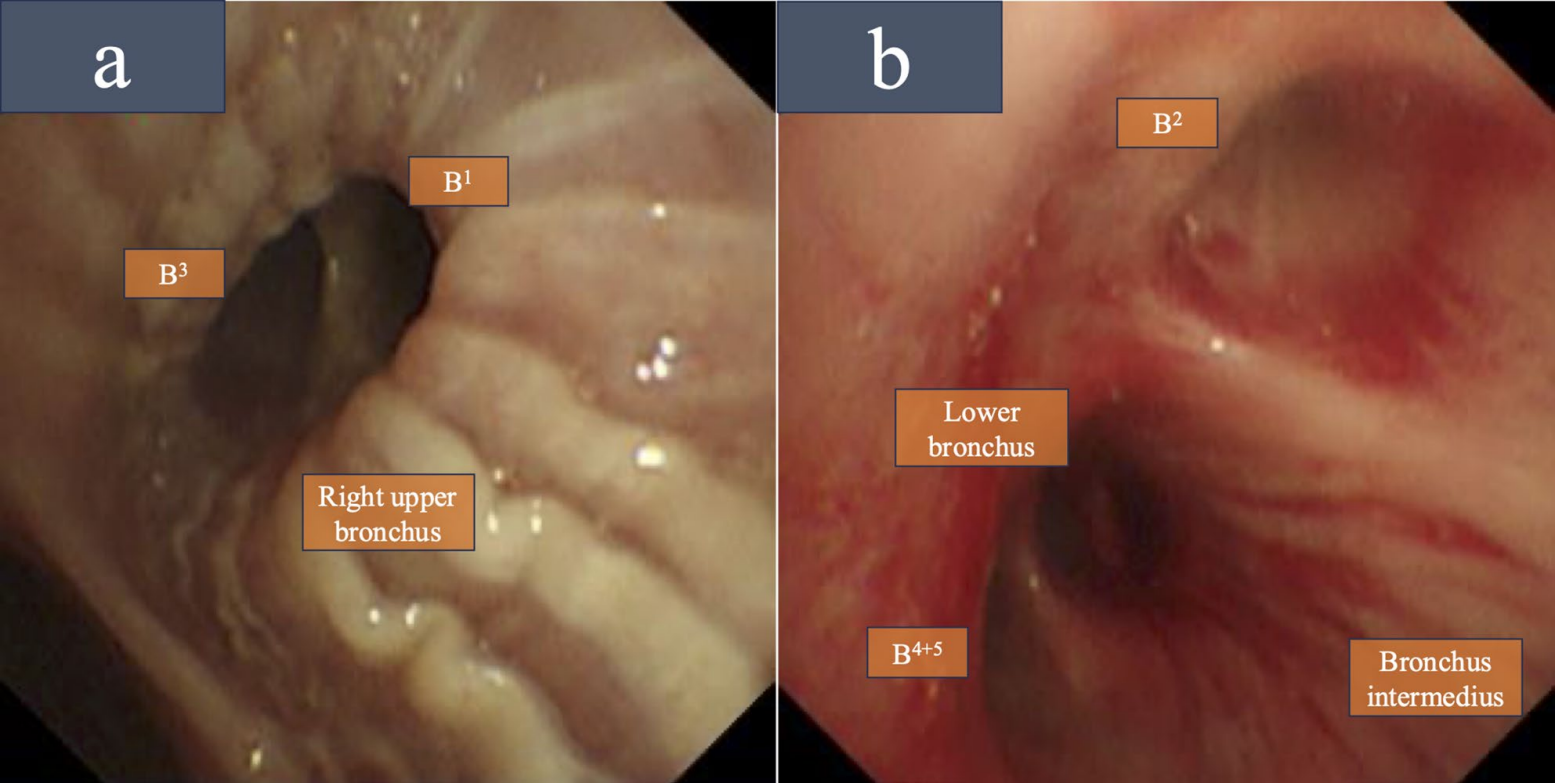
Preoperative bronchoscopic findings. **a** The B1 + 3 bronchus (right upper bronchus) originated from the right main bronchus. **b** The displaced B2 bronchus arose from the bronchus intermedius

A 73-year-old woman presented to a local clinic with worsening dizziness due to sequelae of a prior cerebral infarction. An incidentally obtained chest CT scan revealed a cavitary nodule in the right upper lobe (RUL), and she was referred to our hospital for further evaluation. Chest CT showed a partially solid cavitary nodule measuring 12 mm in the right upper lobe, with a solid component diameter, 5 mm; consolidation-to-tumor ratio, 0.42, at the border between segments S1 and S3 (Fig. 1a). ^18^F-fluorodeoxyglucose positron emission tomography/CT (¹⁸F-FDG PET/CT) showed FDG uptake in the nodule, with SUV_max_ of 7.4 (Fig. 1b). Contrast-enhanced CT of the chest and abdomen revealed no other tumors. Bronchoscopy confirmed that the displaced B2 bronchus arose from the bronchus intermedius; i.e., proximal to the lower lobe bronchus, whereas a common B1 + 3 bronchus (right upper bronchus) arose from the right main bronchus (Fig. 2a, b). Notably, the displaced B2 bronchus could also be identified on coronal chest CT (Fig. 1c). Transbronchial lung biopsy from the B3a bronchus yielded a diagnosis of squamous cell carcinoma.

**Fig. 3:**
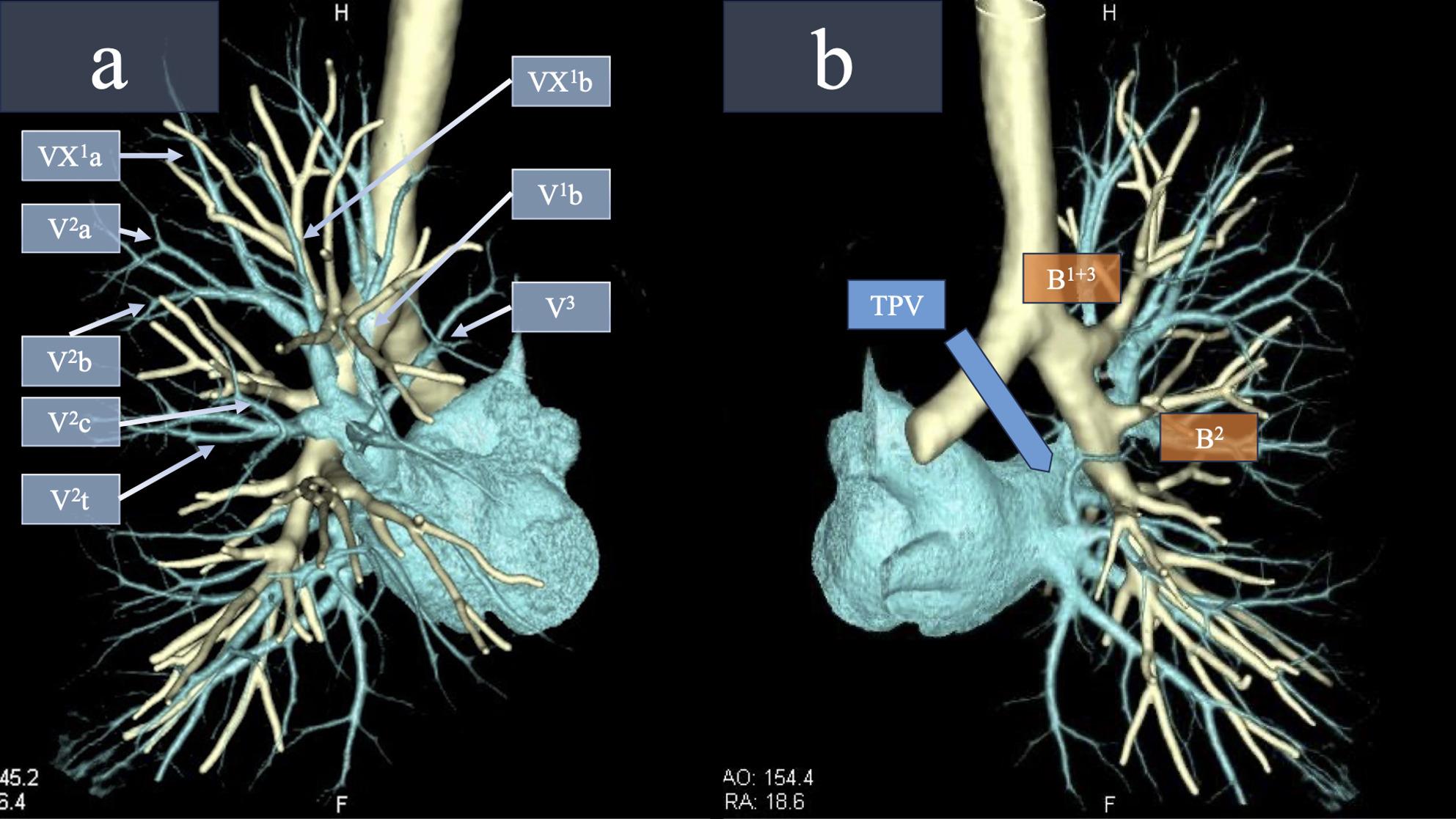
Three-dimensional CT bronchography and angiography (3D-CTBA), venous phase, for preoperative anatomical planning (Ziostation REVORAS; Ziosoft Inc., Tokyo, Japan). **a** In addition to V1b, two veins from segment S1 (VX1a and VX1b) drained into the central vein. **b** The top pulmonary vein was formed by a portion of V2 draining into V6

**Fig. 4:**
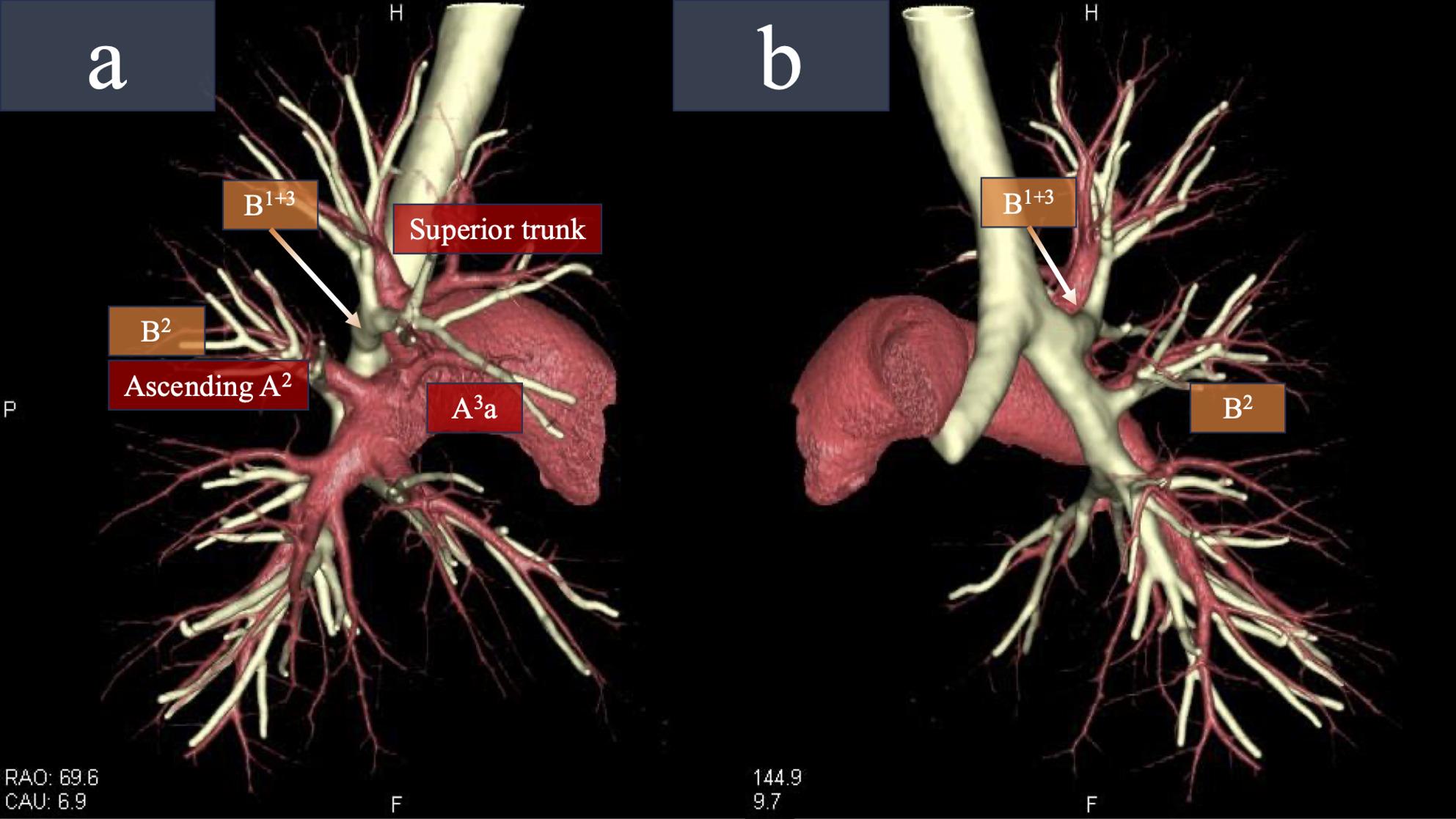
Three-dimensional CT bronchography and angiography (3D-CTBA), arterial phase, for preoperative anatomical planning (Ziostation REVORAS; Ziosoft Inc., Tokyo, Japan). **a** No recurrent A2 branch arose from the superior trunk (A1 + A3b). **b** The displaced B2 bronchus arising from the bronchus intermedius was clearly visualized

3D-CTBA revealed a pattern resembling the Ib (anterior + central vein) type of the right upper lobe pulmonary vein (PV) [4]; however, this pattern differed in that, in addition to V1b draining into the superior pulmonary vein (SPV), two veins from S1 (VX1a and VX1b) drained into the central vein (Fig. 3a). Furthermore, a portion of V2 drained into V6, constituting the top pulmonary vein (TPV) (Fig. 3b). A3a arose from the interlobar pulmonary artery (PA), the ascending A2 branched from its caudal side, and no recurrent A2 branch originated from the superior trunk (A1 + A3b) (Fig. 4a and b). 3D-CTBA also showed that the displaced B2 bronchus arose from the bronchus intermedius, as confirmed by bronchoscopy (Figs. 3b and 4a and b). Thus, the diagnosis was primary lung squamous cell carcinoma (cT1bN0M0, stage IA2) in the RUL at the border between segments S1 and S3 associated with a displaced abnormal bronchus. Laboratory testing showed an elevated serum CYFRA21-1 level of 8.5 ng/mL, with no other abnormal findings. The patient’s comorbidities included sequelae of prior cerebral infarction and bronchial asthma. Pulmonary function testing demonstrated obstructive ventilatory impairment, with an FEV1.0% of 64.05%, whereas the remaining parameters were within normal limits. Taken together with the results of the other preoperative examinations, these findings indicated that the patient was an acceptable candidate for surgery.

**Fig. 5:**
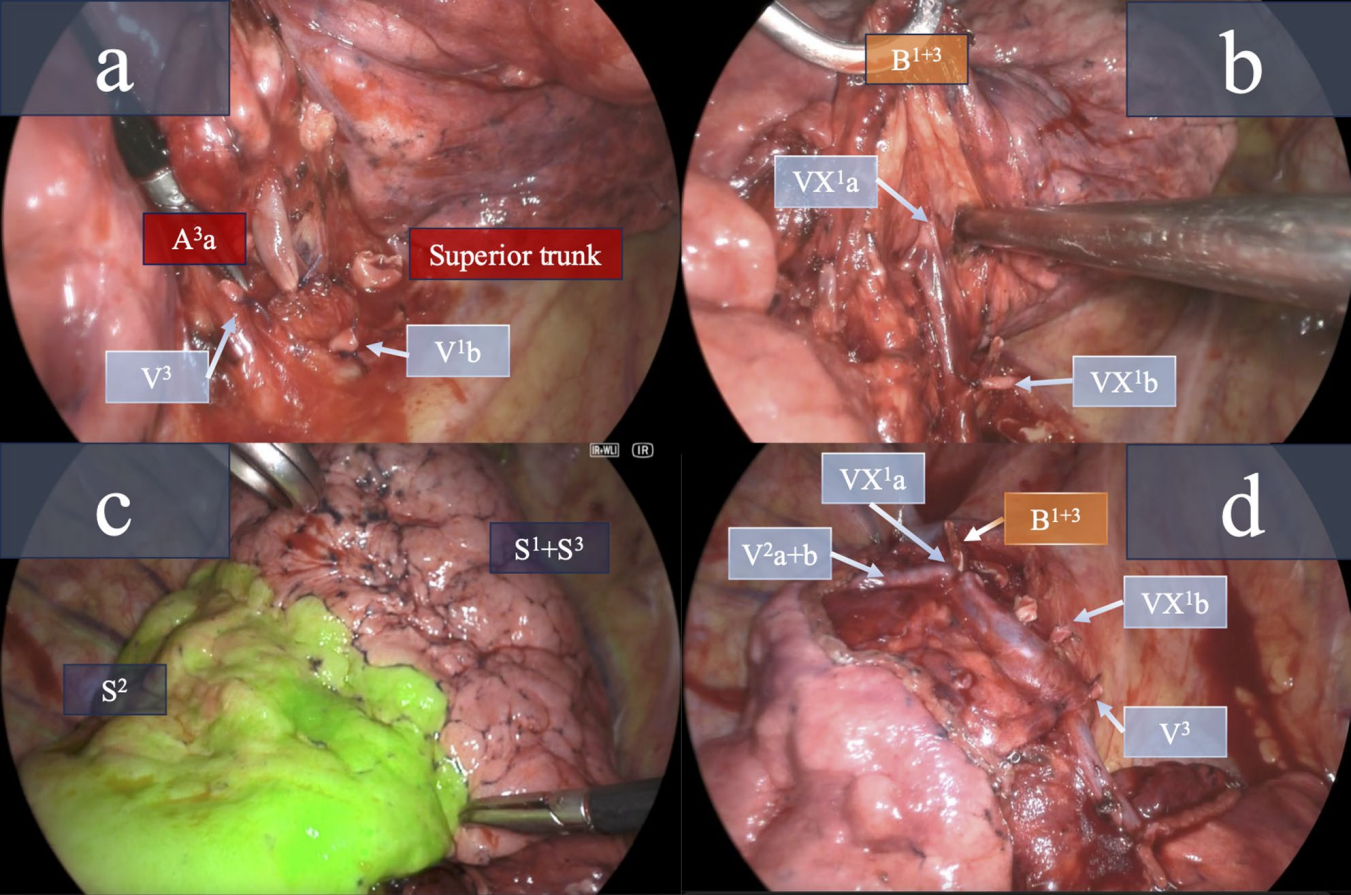
Surgical steps of right S1 + S3 segmentectomy. **a** Division of V1b, V3, and the superior trunk, and exposure of A3a at the hilum. **b** The peripheral stump of B1 + 3 was retracted dorsally, allowing division of VX1a and exposure of VX1b draining segment S1 into the central vein at the hilum. **c** Identification of the intersegmental plane after intravenous injection of indocyanine green (ICG). **d** Division of the intersegmental plane with an endostapler, completing the S1 + S3 segmentectomy

Considering the tumor size and consolidation-to-tumor ratio (CTR), and with reference to the Japanese Clinical Practice Guidelines for Lung Cancer (2025 edition) [5], right S1 + S3 segmentectomy with hilar lymph node dissection was performed via three-port VATS [Additional file 1, Additional file 2]. Intraoperatively, the fissures between the upper and middle lobes and between the upper and lower lobes were both incomplete. V1b was ligated and divided at the ventral aspect of the pulmonary hilum, after which the superior trunk was dissected, ligated, and divided. V3 draining into the central vein was ligated and divided. A3a, which arose from the interlobar PA, was also dissected, ligated, and divided (Fig. 5a). Hilar lymph nodes were carefully dissected. The peripheral stump of A3a was retracted dorsally, and the posteriorly located B1 + 3 was encircled and then divided with an endostapler, after confirming its identity by intraoperative bronchoscopy. The peripheral stump of B1 + 3 was retracted dorsally, and the VX1b and VX1a veins draining segment S1 into the central vein were ligated and divided, completing the hilar dissection (Fig. 5b). The interlobar fissure between the upper and middle lobes was then divided with an endostapler. Using indocyanine green (ICG) (Fig. 5c), the intersegmental plane was identified and divided with an endostapler, thereby completing the S1 + S3 segmentectomy (Fig. 5d). The hilar lymph node around the B1 + 3 bronchus, corresponding to station #12u, was submitted for intraoperative frozen-section examination, which showed no metastasis; therefore, the procedure was completed with hilar lymph node sampling.

The operative time was 3 h and 7 min, and the intraoperative blood loss was 40 mL.

The postoperative course was uneventful. The chest tube was removed on postoperative day 3. The pathological diagnosis was squamous cell carcinoma, pT1bN0M0 stage IA2, with a pathological surgical margin of 2.7 cm.

## Discussion and conclusions

Several classification systems for bronchial anomalies have been proposed [1, 2, 6], and their reported frequencies are broadly consistent, with an overall prevalence of tracheobronchial branching anomalies of 0.64–0.76%, and RUL anomalies accounting for 75.3% of all cases. An independent origin of the right B2 bronchus from the bronchus intermedius is extremely rare, having been reported in only 7 of 6,480 cases (prevalence 0.1%).

To our knowledge, lung cancer cases complicated by a displaced right B2 bronchus have been described in only eight previous reports (Table 1) [7–14].

**Table 1:** Reported cases of right upper lobe lung cancer with displaced B^2^

Author (year)	Age/sex	Tumor location	LNM/Stage	Surgical procedure	Major fissure	TPV	PV
Ohsumi A (2010) [7]	56/M	S1	Negative/IA3	Lobectomy (VATS)	Incomplete	-	V1-V3; Anterior Vein + Central Vein
Sakaguchi K (2013)[8]	83/M	S1	Negative/IB	Lobectomy (VATS)	Incomplete	-	V1-V3; Anterior Vein + Central Vein
Tatematsu T (2016) [9]	72/F	S1	Negative/IA2	S1 + S3 segmentectomy (VATS)	Incomplete	-	V1-V3; Anterior Vein + Central Vein
Onodera K (2017) [10]	66/F	S1	Negative/IA2	Lobectomy (open)	N/R	-	V1-V3; Anterior Vein + Central Vein
Momose N (2020) [11]	73/F	S2	Negative/IA2	Lobectomy (VATS)	Incomplete	+	V1-V3; Anterior Vein + Central Vein V2; dorsal to the intermediate bronchus, draining V6 (IPV)
Sato F (2021) [12]	67/M	S1/S3	Negative/IA2	Lobectomy (open)	Incomplete	-	V1-V3; Anterior Vein + Central Vein
Konno H (2023) [13]	73/F	S2	Positive/IIB	Lobectomy (VATS)	N/R	+	V1-V3; dorsal to the PA, draining into the SPV
Imanaka T (2025) [14]	66/F	S2	Negative/IA2	S2 segmentectomy (open)	Incomplete	-	V1-V3; Anterior Vein + Central Vein
Present case	73/F	S1/S3	Negative/IA2	S1 + S3 segmentectomy (VATS)	Incomplete	+	V1-V3; Anterior Vein + Central Vein V2; dorsal to the intermediate bronchus, draining V6 (IPV) VX1a, VX1b

*LNM* lymph node metastasis, *VATS* video-assisted thoracoscopic surgery, *TPV* top pulmonary vein

^*^ N/R: not reported in the original article

Among these, to the best of our knowledge, only the case reported by Tatematsu et al. described an S1 + S3 segmentectomy in a patient with a displaced B2 bronchus. Furthermore, no previous report has clearly demonstrated the anomalous pulmonary venous course using both preoperative three-dimensional computed tomography and intraoperative images, including surgical video documentation. Therefore, the present case may provide useful anatomical and surgical insights for future lobectomy or segmentectomy in patients with a displaced B2 bronchus.

One notable feature observed in these cases is the presence of an anomalous course of the TPV, defined as a portion of the right SPV passing behind the bronchus intermedius [11, 13, 15]. In the present case, the pulmonary venous anatomy corresponded to that of the TPV. However, given the chosen surgical procedure, this anomalous course did not affect the operative maneuvers. Furthermore, the pulmonary venous pattern was such that V1b drained into the SPV, and two additional veins from S1 (VX1a and VX1b) drained into the central vein; this configuration may not fit any of the types described in the classification proposed by Shimizu et al. [4].

Preoperative assessment of pulmonary venous anatomy is important to prevent misidentification of vessels, avoid unexpected bleeding from unanticipated branches, and help ensure an adequate surgical margin, which is a critical issue in segmentectomy.

Pulmonary arterial anomalies have generally been considered to be absent in right B2-type displaced bronchi, but Tatematsu et al. [9] demonstrated their existence, specifically describing aberrant arterial branching in which A1a and A2a arose from A6 and A2b arose from A4 + 5. Moreover, many reported cases, including the present case, have an incomplete major fissure, which may represent another shared characteristic among these cases [2].

Although preoperative 3D-CTBA and ICG fluorescence imaging are not novel techniques themselves, their case-specific application was useful in the present patient for understanding the unusual bronchovascular anatomy and planning a safe anatomical segmentectomy. If a displaced B2 bronchus is confirmed preoperatively, two practical points require emphasis: (i) the presence of TPV should be carefully assessed on preoperative 3D-CTBA, and (ii) if an incomplete major fissure is present, an appropriate surgical strategy (e.g., a fissureless approach, including the “no-touch fissure” technique [16, 17]) should be planned in advance to avoid misidentification of bronchovascular structures and inadvertent bleeding. These points represent practical pitfalls specific to this anomaly and should be recognized before surgery.

Randomized trials have shown that segmentectomy is non-inferior to lobectomy for early-stage lung cancer [18, 19], and thus, segmentectomy has increasingly been adopted as a standard surgical treatment for lung cancer. In previous reports of lung cancer associated with displaced B2, right upper lobectomy has been the main surgical procedure. However, as illustrated by the case reported by Imanaka et al. [14] and by the present case, segmentectomy may be a feasible parenchyma-sparing option in carefully selected patients, depending on tumor location and clinical stage, including nodal status. In the present case, the tumor was small and clinically early-stage, and an adequate pathological margin of 2.7 cm was achieved.

At our institution, when a lesion is considered to have relatively aggressive biological behavior, we generally prefer upper lobectomy rather than segmentectomy, even when an adequate surgical margin can be achieved for the primary tumor, because of the potential presence of occult intrapulmonary metastasis. Therefore, if the present patient had had right upper lobe lung cancer with normal anatomy and without a displaced B2 bronchus, we would likely have selected right upper lobectomy. In the present case, however, based on the tumor location, we considered that the tumor was unlikely to be closely related to the lymphatic drainage pathway along the displaced B2 bronchus. From an oncological perspective, we therefore considered right S1 + S3 segmentectomy to be an oncologically reasonable alternative to right upper lobectomy in this specific anatomical setting, and judged that additional resection of S2 would provide limited oncological benefit. Intraoperative assessment of nodal status was therefore essential. In this case, the lymph nodes around B1 + 3 were submitted for intraoperative frozen-section examination, and no nodal metastasis was identified. In addition, the patient had asthma as a comorbidity, and preservation of pulmonary function was considered clinically important.

Although hilar lymph node sampling with intraoperative frozen-section examination was negative in the present case, occult mediastinal nodal metastasis cannot be completely excluded, particularly in lesions with potentially aggressive biological behavior, and this remains an important oncological limitation of segmentectomy. Thus, lobectomy should still be preferred when the tumor is suspected to have a higher malignant potential, when nodal involvement cannot be confidently excluded, or when an adequate surgical margin is not expected.

Therefore, even in the presence of marked bronchovascular anomalies, segmentectomy may be considered in highly selected patients with small, early-stage tumors when an adequate surgical margin is expected, nodal status is carefully evaluated, and meticulous preoperative assessment and precise intraoperative recognition of the bronchovascular anatomy are achieved.

An additional advantage of segmentectomy is preservation of pulmonary function. In the present case, the measured FEV1 at 7 months postoperatively was 1.29 L, which was slightly lower than the predicted postoperative FEV1 after right S1 + S3 segmentectomy (1.39 L). However, this finding does not negate the rationale for parenchyma-sparing resection in the present case. Rather, because the predicted postoperative FEV1 after right upper lobectomy was even lower (1.31 L), a more extensive resection would likely have resulted in further functional loss. In addition, volumetric assessment of the lung parenchyma using Ziostation REVORAS (; Ziosoft Inc., Tokyo, Japan) showed that the volumes of S1 + 3 and S2 were 0.738 L and 0.337 L, respectively, with a ratio of 2.2:1. This finding suggests that preservation of S2 may still contribute to parenchymal preservation, even though the expected functional gain was modest. Given that additional resection of S2 was considered unlikely to provide substantial oncological benefit in this specific anatomical setting, limiting the resection to S1 + S3 remained a reasonable strategy.

This report is limited by the fact that it describes a single case, and only a small number of published cases are available for comparison. Definitions and descriptions of TPV and related venous variants also vary across reports, which may limit direct comparability. Further accumulation of cases using standardized 3D-CTBA-based evaluation may help refine classification systems and identify clinically relevant red flags and operative decision points in patients with displaced right B2 bronchi.

In conclusion, we describe a rare case of RUL lung cancer with a displaced right B2 bronchus, TPV, and atypical segmental venous branching, successfully treated by VATS right S1 + S3 segmentectomy. Careful preoperative assessment with bronchoscopy and 3D-CTBA, together with intraoperative guidance using standard adjunctive techniques, enabled safe anatomical resection by clarifying the complex bronchovascular anatomy and securing an adequate margin. This case suggests that anatomical segmentectomy can be a feasible parenchyma-sparing option for selected early-stage tumors adjacent to bronchial anomalies, provided that careful preoperative planning and meticulous intraoperative execution are achieved.

## Supplementary Information

Supplementary Material 1.

Supplementary Material 2.

Supplementary Material 3.

## Data Availability

The datasets supporting the conclusions of this article are included within the article and its supplementary video file.

## Abbreviations

3D-CTBA: three-dimensional computed tomography bronchography and angiography
CT: computed tomography
FDG: fluorodeoxyglucose
ICG: indocyanine green
PA: pulmonary artery
PET: positron emission tomography
PV: pulmonary vein
RUL: right upper lobe
SPV: superior pulmonary vein
TPV: top pulmonary vein
VATS: video-assisted thoracoscopic surgery
